# Implementation of Context Aware e-Health Environments Based on Social Sensor Networks

**DOI:** 10.3390/s16030310

**Published:** 2016-03-01

**Authors:** Erik Aguirre, Santiago Led, Peio Lopez-Iturri, Leyre Azpilicueta, Luís Serrano, Francisco Falcone

**Affiliations:** 1 Electrical and Electronic Engineering Department, Public University of Navarre, Pamplona 31006, Spain; erik.aguirre@unavarra.es (E.A.); santiago.led@unavarra.es (S.L.); peio.lopez@unavarra.es (P.L.-I.); lserrano@unavarra.es (L.S.); 2 School of Engineering and Sciences, Tecnologico de Monterrey, Monterrey 64849, Mexico; leyre.azpilicueta@itesm.mx

**Keywords:** social sensors, wireless body area networks, deterministic radio planning, back office

## Abstract

In this work, context aware scenarios applied to e-Health and m-Health in the framework of typical households (urban and rural) by means of deploying Social Sensors will be described. Interaction with end-users and social/medical staff is achieved using a multi-signal input/output device, capable of sensing and transmitting environmental, biomedical or activity signals and information with the aid of a combined Bluetooth and Mobile system platform. The devices, which play the role of Social Sensors, are implemented and tested in order to guarantee adequate service levels in terms of multiple signal processing tasks as well as robustness in relation with the use wireless transceivers and channel variability. Initial tests within a Living Lab environment have been performed in order to validate overall system operation. The results obtained show good acceptance of the proposed system both by end users as well as by medical and social staff, increasing interaction, reducing overall response time and social inclusion levels, with a compact and moderate cost solution that can readily be largely deployed.

## 1. Introduction

Population ageing is unprecedented, without parallel in human history—and the twenty-first century will witness even more rapid ageing than did the century just past [1]. This global phenomenon is affecting the whole world, although with different evolution rates of the process, depending on regions or countries. In any case, the main goal for the future is to ensure people everywhere will be ageing actively, making possible their participation in social activities without any restrictions.

Hence, to reach the above challenge, an embedded Social-Health care action or strategy where citizen empowerment should be the solution’s central point is mandatory [2]. This strategy must take into account all personal and context factors such as personal health, work and economic situation, social networks, *etc.* that affect active aging and assisted living processes. This Social-Health care strategy should be based on massive use of Information and Communication Technologies (ICT) for making possible a service deployment with several main features as cost-effective, plug and play operation, minimal user’s intervention, and helpful for the largest number of citizens.

Adopted ICT Social-Health care solutions could be included in two possible scenarios [3]. On the one hand, indoor monitoring scenarios, so-called home monitoring is defined where the user’s state is controlled at his or her own residence. These scenarios provide more real information about the user due to the fact that the remote control process is achieved at a comfortable and regular environment. On the other hand, outdoor monitoring scenarios that include all situations where the user is away from home (office, walking on street, gym, *etc.*) and is also continuously monitored. These scenarios provide mobility to the user while the control process is performed. In both scenarios, the devices (for medical or behavioral monitoring), are portable or wearable, general purpose, and user-friendly. Moreover, they are equipped with short range (Bluetooth, Zigbee, IrDA, *etc.*) and/or large range (GSM-GPRS, UMTS, *etc.*) wireless technologies.

Currently, the use of Wireless Sensor Networks (WSN) continues to grow in a wide variety of application fields [4,5,6], such as agriculture and farming [7], infrastructure state monitoring [8], location and guiding [9], vehicular communications [10] and healthcare monitoring [11,12], to name a few. The future trend seems to be the increase of the number of wireless nodes in order to collect more information from the surrounding environment, bringing the Internet of Things (IoT) to our daily life.

Among WSNs, Mobile Ad-hoc Networks (MANET) have attracted the attention of many research groups around the world, becoming popular due to the unique characteristics they provide: wireless mobile devices with limited resources that can exchange information with each other without any fixed infrastructure. Thus, MANETs provide easier deployment, system maintenance and upgrade. These characteristics make MANETs an adequate solution to solve efficiently many applications, as is the case of the telemedicine and healthcare system presented in this work. In fact, in the literature can be found several works of MANETs applied to healthcare environments, such as hospital and big in-building environments [13], tele-emergency projects [14], tele-care and telemedicine systems [15,16], real time medical data acquisition and patient monitoring systems [17,18,19], and more specific applications such as tele-cardiology [20], emergency telemedicine system in disaster areas [21] and monitoring combat soldiers [22].

In Spain, the Social-Sanitary System term (in Spanish “*Sistema Socio-Sanitario*”) makes reference to the public or private healthcare system focused on guaranteeing the best population’ state regarding general aspects as life quality, well-being, and social integration. Meanwhile, the Sanitary term makes reference to the healthcare system part responsible for solving physiological and psychological problems suffered by the population. The Social term includes all the aspects associated with population social problems such as loneliness, integration into the society, energy poverty, population ageing, among others; problems that require specific healthcare professionals like social workers or assistants. The Social-Sanitary System concept is deeply adopted by Spanish population.

Social problems suffered by population can be detected and monitored in the same way as clinical diseases. Thus, it is required to know the user’s social state by means of specific devices and sensors responsible for acquiring outstanding parameters associated to daily activity, habits, and in general any information that makes possible to work a social problem out. In this sense, environment measurements are combined with information related with user behavior, providing additional insight into the socio-sanitary context of the user; e.g., water consumption, electricity and gas consumption, operation time of HVAC (heating, ventilating and air conditioning) system, open/close status of doors and windows, among many others. Thus, water consumption can provide information about frequency the user goes to the toilet or takes a shower. A very low consumption can indicate the user suffers some degenerative disease or poverty situation. Electricity/gas consumption together with operation time of HVAC system can indicate the user is in energy poverty situation; if the user does not have enough resources for operating HVAC system during a suitable time period, the consumption values will be very low compared to average value. Because all these problems have a social nature and they must be treated by social workers and assistants, the sensors used to acquire their associated information are called social sensors throughout this paper.

In general, social sensors are environmental acquisition devices that require minimum user intervention, and for this reason, they are placed at previously established locations; ceiling for temperature/humidity sensors, doors and windows for open–close detection, sink pipe for water consumption sensors, *etc.* Social sensors exhibit reduced size, wireless communication capabilities, and minimum current consumption allowing them to be battery powered and highly operation autonomous; this feature allows the reduction of maintenance service costs. Moreover, social sensors must ensure the lowest visual and structural impact when embedded within the planned service scenario. Personal health devices show features similar to social sensors. However, they do have greater variety concerning form factor and location within the monitoring scenario. Some health devices like weigh scale, blood pressure monitor, and glucose meter could be used only a few times every day and they do not require to be worn by the user all the time. Thus, these devices could be portable sensors always located at the same living space, mainly bathroom and bedroom; when a biomedical measurement must be taken, the user goes to the living space where the medical device remains and uses it. Other health devices like electrocardiogram monitors require a real time monitoring process and they must be worn by the user. These devices must be wearable sensors and their location inside the monitoring scenario is not fixed, but changes as the user moves.

With regards to ICT, there is no implementation difference between health and social sensors because they require similar functional blocks: analog front-end (AFE), A/D conversion, microprocessor, power supply, and wireless communication. The resources provided by these blocks are conveniently used according to the sensor type, intelligent-processing needs, autonomy requirements, *etc.* This solution is currently being used for ICT Social-Health care service in Navarra (in Spanish “*Navarra-ASISte-TIC*”, NASISTIC) project deployment [22] as a proof of concept of an integral Social-Healthcare system monitored by Red Cross in Navarra, and focused mainly on providing high-quality services to vulnerable citizens like the elderly or people suffering mental illnesses. Moreover, Navarra region shows an exceptional scenario for this deployment because it includes populations living in a medium-size city, Pamplona, as well as sparse citizens living in rural and mountain surroundings.

The novelty of the proposal presented within this work is focused in the development of Integrated Socio-Sanitary Services based on Information and Communication Technologies and with minimal user interaction, providing integral user care given by inherent interdependence between Health and Social Environment. In the context of elderly persons, a main target of the system (with elements such as dependence, loneliness, assisted living, *etc.*), this combined rollout is of particular importance, because degradation of health state can be caused by the degradation of the social context (energetic poverty, poor nutrition, cleanliness, *etc.*) and *vice versa*. In this way, deployments such as NASISTIC are of special interest in order to provide integral control of elderly people, people in risk of social exclusion, chronic patients, among others.

In this work, the implementation of Social Sensor devices and their application to e-Health monitoring within the framework of an urban scenario, given by project NASISTIC in Navarra, Spain, will be analyzed. Different considerations of physical implementation of the devices, considering multiple signal capability as well as usability factors (such as ergonomics and power consumption) will be analyzed. The usability and performance of the devices are strongly dependent on the behavior and influence of wireless transceivers, which are analyzed by deterministic techniques, providing assessment in the configuration of individual nodes as well as in the location and number of transceivers as a function of the scenario. The developed sensors will then be tested in a living lab configuration, which serves as the base for a future full-scale deployment.

The paper is structured as follows: Section 2 describes the architecture and functionality of the Social Sensor Devices, based on a multi signal Input/Output controller in which different sensors can potentially be implemented. Section 3 analyzes the impact of the wireless channel behavior, which is a key parameter to evaluate overall performance of the Social Sensor devices in terms of mobility, security and quality of the exchanged information, as well as on the design of individual nodes and layout of node network architecture. In Section 4, initial tests within a Living Lab framework are described, providing insight in complete system operation and the potential full-scale deployment. Finally, Section 5 provides conclusions and final remarks of the work.

## 2. Related Work

Different solutions based on multiple sensor platforms and configurations have been proposed in order to implement remote health-monitoring, diagnostics and medical services, with special interest in the past decade [23,24,25], due to their capability of reducing overall costs and increasing quality of life metrics. Solutions have been provided in the context of application of Information and Communication Technologies in order to allow system interoperability and enhance medical service, by means of solutions such as electronic patient records or digital image records, to name a few. A step further is achieved by adding mobility to previous e-Health scenarios, by means of integration of mobile terminals, connected to Public Land Mobile Networks (PLMN), allowing initial tele-monitoring capabilities. Other solutions, such as advanced care and alert portable telemedical monitor (AMON) project [26] employed GSM transmission links to communicate a wrist wearable device, with capability of measuring multiple signals, such as Electrocardiograms (ECG), Electromyograms (EMG), location or skin conductance. Cordless phones have also been employed as transmitter device, to which a specialized sensor unit was connected, implementing a real time wireless physiological monitoring system [27]. Prior to the popularization of Smart Phones, several solutions employed Personal Digital Assistants (PDA), with basic control functions of multiple sensor devices connected to sensor boards [28].

One of the main drivers in order to increase system interactivity is the use of compact size biomedical sensors, which can communicate in real time with medical specialists or social services, or can perform data-logging functions in order to store and send the relevant information later. In this sense, communication systems shift from PLMN based systems to Wireless Personal Area Network/Body Area Network devices, with predominance in the use of 802.15.4 standards, such as Bluetooth or ZigBee [23]. The main benefit in this approach is the inherent capability of connecting multiple devices, small size and reduced energy consumption as compared with PLMN/Wireless Local Area Network (WLAN) solutions.

Another relevant aspect in the adoption of remote health monitoring solutions is the advance in the implementation of sensors as well as the capability of embedding them in truly wearable configurations. In this sense, it is possible to measure multiple bio-physical parameters, such as glucose levels, blood pressure, oxygen saturation, respiration rate, ECG, and EMG, which can be combined with environmental parameters and user movement and location. The use of the combined information of these multiple sensors and sources leads to truly context aware Ambient Assisted Living (AAL) scenarios, with multiple solutions reported [23]. A following step in system integration is embedding sensors in textiles within user garments, in order to increase ergonomics as well as in depth user monitorization. Different solutions have been proposed, such as the MagIC vest or the MyHeart instrumented shirt, in which multiple sensor elements have been included within the clothing [29,30,31,32,33]. As a further step, specific software development frameworks, such as SPINE, have been proposed and multiple Body Sensor Network applications have also been proposed, such as rehabilitation, Gait analysis, emotional stress detection or handshake detection, to name a few [34]. Table 1 presents a comparison of different remote health monitoring systems.

## 3. Social Sensor Devices

In order to analyze the wireless communication in a generic social-sanitary monitoring system and emulate the operation of any acquisition sensor, a specific evaluation system has been developed. The system consists of two basic Bluetooth wireless modules: transmitter and receiver. The first one represents the health/social sensor, and it is placed at different locations inside the monitoring scenario in order to simulate the behavior and communication process of a real system. The second one represents the gateway device responsible for gathering the information acquired by sensors. Although this device could be implemented in a real smartphone or tablet platform, most of indoor scenarios include a set-top box for receiving data. For this reason, the receiver in the evaluation system is placed on a fixed location while the transmitter is moved around the scenario. Several wireless transceiver technologies have been analyzed and tested, mainly in the Wireless Body Area Network (WBAN)/Wireless Personal Area Network (WPAN) context. The election of Bluetooth allows inherent integration of mobile terminals such as smartphones within the Social Sensor scenario, enabling a wide variety of applications and functionalities which can be used by the end user, as well as by medical staff, social working staff and technical staff, increasing overall interaction and service provision.

**Figure 1:**
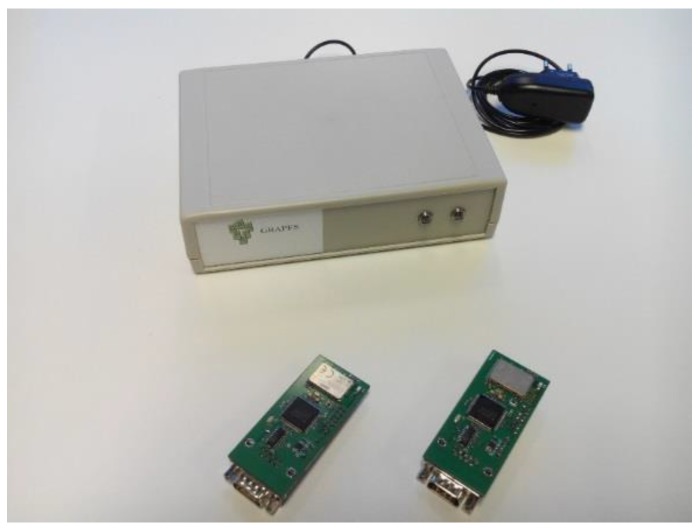
Evaluation module of the implemented Social Sensor device and a Home Hub.

Transmitter and receiver are *Ad-Hoc* evaluation modules connected to a PC or Laptop via serial port, which allow the execution of two relevant tasks. On the one hand, the configuration of data chunk size and transmission period are used by sensor module. By modifying these transmission parameters, it is possible to emulate the communication behavior of any acquisition sensor; from devices which transmit huge amounts of information in a continuous way being the case of real-time/event-driven electrocardiogram monitors, to devices with low data size and transmission rate requirements just like blood pressure monitors or humidity sensors. On the other hand, this allows the reception of link quality measurements. The receiver module provides information about received power in form of Received Signal Strength Indication (RSSI) levels and communication channel quality by means of Bit Error Rate (BER) values. These parameters are really useful in the radio propagation study. Figure 1 shows implemented evaluation modules as well as a final social sensor hub device.

**Figure 2:**
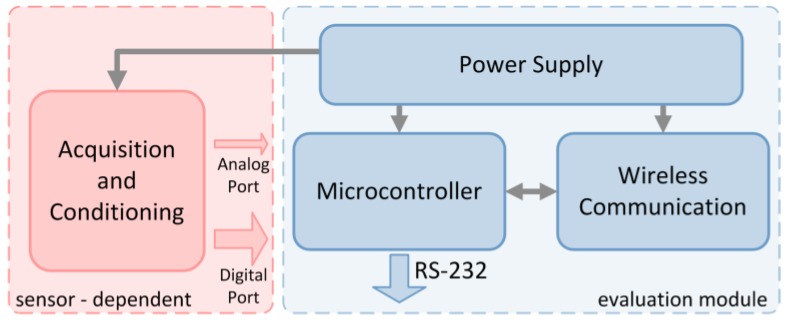
Evaluation Module architecture.

The evaluation module contains several functional blocks (Figure 2). These blocks have been designed and implemented according to very low power consumption and reduced form factor requirements. Although the module is an evaluation board focused on providing versatility for modeling any acquisition sensor, the design restrictions mentioned previously allow the evaluation module to get close the features of portable and wearable real sensors. The description of the main functional blocks is the following:

- Wireless communication: This block manages all the Bluetooth communication tasks like device search, connection establishment, flow control, and data transmission, among others. Bluegiga’s WT12 module is used for implementing this block. The module includes the whole protocol stack, application profiles, a virtual machine for execution of software code, and several General Purpose Input/Output (GPIO) terminals. Concerning wireless communication, the evaluation module is a Bluetooth class 2 device with maximum transmission power of 3 dBm and data rates up to 3 Mbps. The application data exchange is based on Serial Port Profile (SPP), which allow the emulation of a serial wired RS-232 communication between transmitter and receiver.
- Microcontroller: This block is based on a 16-bit ultra-low power microprocessor, which achieves WT12 module management, acquired data processing, and serial wired communication. The microcontroller receives RSSI and BER parameters from wireless block and transmits them to Laptop in order to be displayed and/or stored. Any sensor must include a microcontroller block according to the device’s features and processing requirements; weigh scales and open/close sensors could require 8-bit low resources processors while electrocardiogram monitors could be based on 32-bit processors with high CPU clock frequency as well as memory, communication ports, and processing resources.
- Power supply: This block provides to evaluation module the voltage required for operating correctly. Based on high efficiency DC-DC regulators together with Low Voltage-Low Power (LV-LP) integrated circuits, the block generates 3.3 V voltage supply by means of two 1.5 V AAA batteries. The main features of these batteries are reduced size and weight, high capacity, and short-time pulse current support. This power supply configuration is representative of most health and social sensors. Although the use of coin cell batteries in order to reduce device’s form factor could be appropriate, this type of battery is not widely used with Bluetooth classic technology because it does not provide enough capacity.

**Figure 3:**
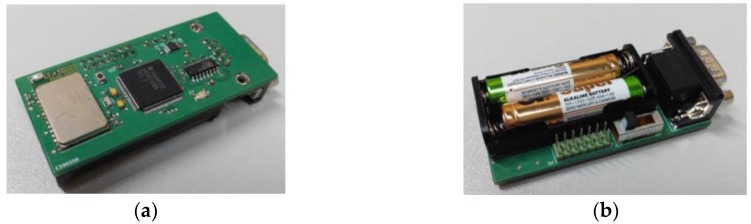
Evaluation module for the Social Sensor devices: (**a**) bottom view; and (**b**) top view.

The evaluation module is depicted in Figure 3. Although the module has been specifically designed for performing radio propagation analysis, it shows features in fact close to real health/social sensors. On the one hand, the module does have reduced size (72.5 mm × 33.0 mm) and weight (24 g) as is required in portable and wearable devices. On the other hand, the module shows high operation autonomy thanks to use LV-LP integrated circuits together with optimized software implementation; in addition, a microcontroller and wireless communication block presents Deep Sleep states that allow reducing the power consumption. All these features make possible to have an evaluation module with features similar to commercial health and social sensors.

**Figure 4:**
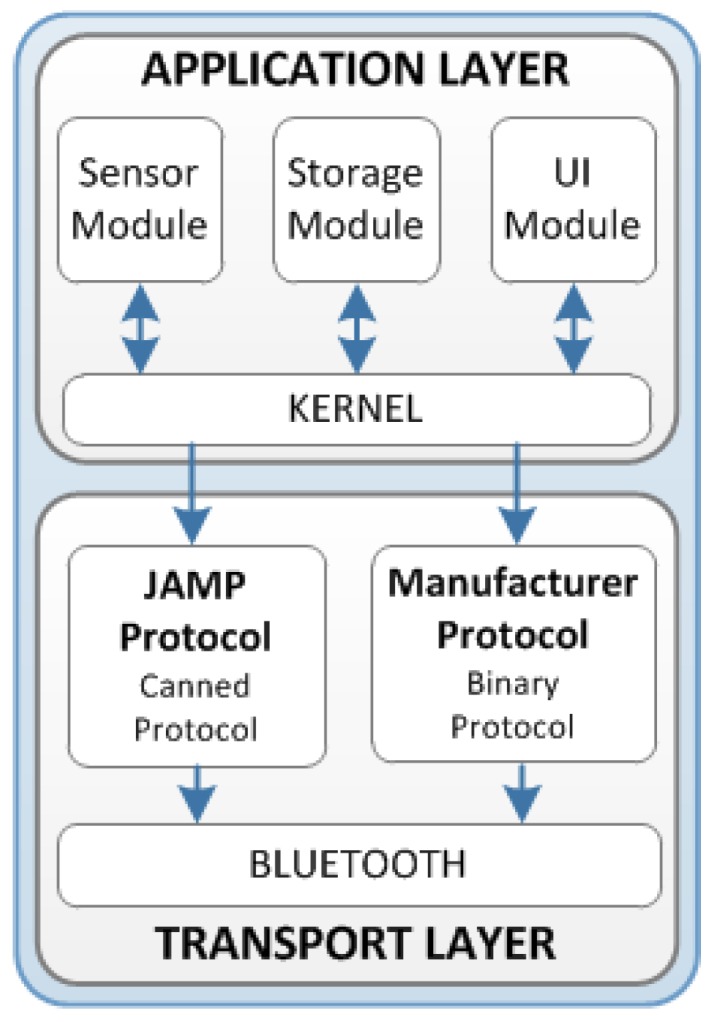
Software architecture for the sensor node devices.

Figure 4 shows a general architecture representative of any device or sensor. According to the device/sensor type, this architecture will include the required software module. The architecture layers are following:

- Transport layer: This layer implements the communication technology used by devices and sensor. In case of NASISTIC project, all devices use wireless Bluetooth technology. This layer also includes the data protocol required for exchange information between devices and set-top box. In this sense, two different protocols are used depending on the type of device or sensor. They are the following:
  - JAMP (*JSON Agent Management Protocol*) is a canned data protocol implemented in the social sensors that have been developed specifically for the project.
  - A manufacturer protocol implemented in medical devices. This is a binary data protocol defined by the manufacturer so it cannot be modified.
- Application layer: This layer can contain different software modules according to the device’s features and functionality. In any case, there is an essential module that performs all the tasks related to measurement gathering; it is the so-called acquisition module. There are other optional software blocks such as User Interface and storage modules. All these modules and the interaction between application and transport layers are managed by a Kernel.

**Figure 5:**
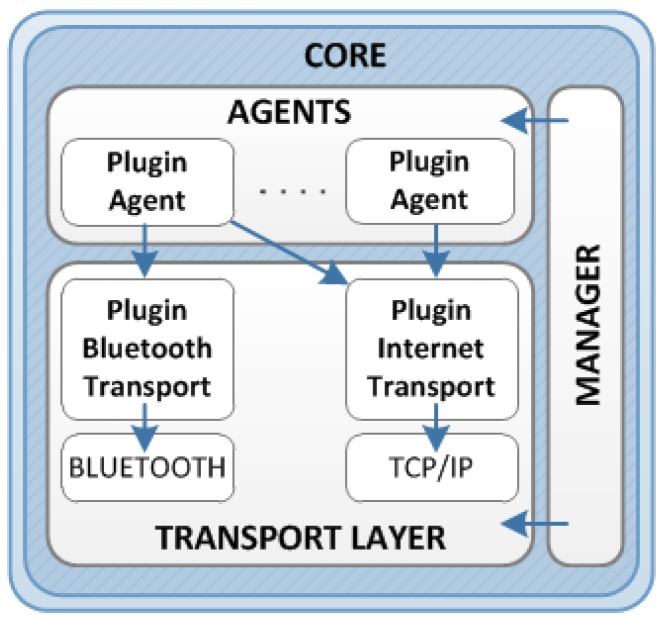
Software architecture for the gateway node device.

In general, the gateway software makes the communication between the sensors and the back-end system easy. The software helps to deal with different transport technologies and protocols in order to gather data from a source (medical devices and social sensors) and deliver it to a destination (back-end system). The software architecture shown in Figure 5 defines the following main elements.

- Core: This is the most important element and includes all the software modules required for the management of layers, and the communication from one layer to each other.
- Plugins: This element represents all the software modules that can be connected to the platform in order to add features. The following are the main plugins.
  - Agents: These plugins are used to wrap connections between source and destination. The agent must be provided with a socket for performing a connection; thus, the socket makes the communication between the device/sensor and the back-end system possible. In addition, the agent provides its own services and methods: installation, open/close a connection, abort a connection, *etc.*
  - Transports: These plugins provide the different sockets required for agents. Obviously, the software architecture is able to use any implemented transport plugin, although NASISTIC software includes the following: Internet transport based on HTTP and TCP/IP protocols, and Bluetooth transport based on bluecove library.
  - Manager: This plugin registers the communication source and destination. These are kept in mind by the client application together its associated active agents. The manager also allows the client to establish a communication with any installed source/destination, or listen to incoming communication through any running transport.

**Figure 6:**
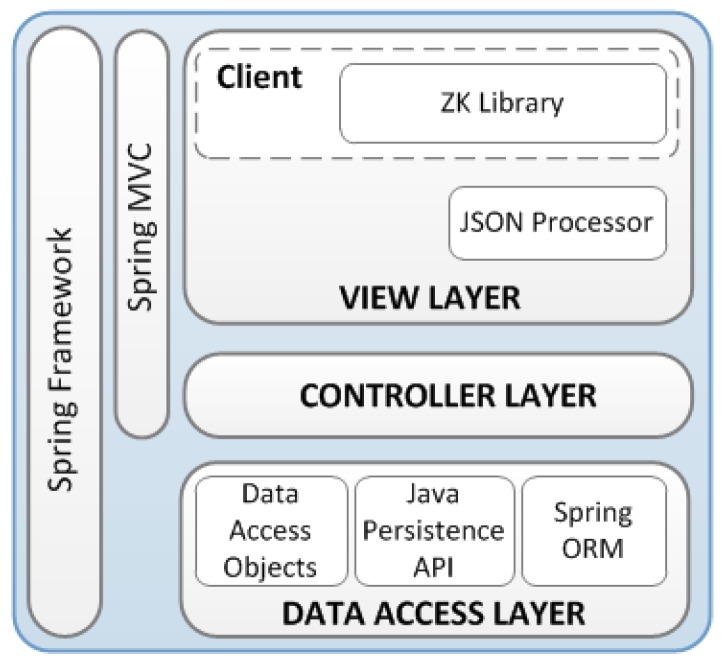
Back-end software architecture.

The back-end’s architecture, depicted in Figure 6, is formed by several logical elements and abstraction layers. The following are the main blocks.

- The Spring Framework and Spring MVC (*Model-View-Controller*) allow the development of flexible and highly connected web applications.
- View layer: This layer provides the user interface according to the client request by means of the ZK library. This library provides an AJAX web framework in order to create a user interface through JAVA programming. In this way, it is possible to develop an environment with RIA (*Rich Internet Application*) features. The view layer also performs the data transmission to final client. The format used in the transmitted information is JSON (*JavaScript Object Notation*).
- Controller layer: It validates the data received from client, and selects the appropriate view for showing them.
- Data access layer: This layer is based on DAO (*Data Access Object*) elements and consists of two main logical blocks: the Java Persistence API focused on the management of persistence and objects mapping, and the Spring-ORM (*Object Relational Mapping*) module, which performs ORM container tasks.

The architecture also includes a web-service layer for showing the measurements acquired by sensors. Afterwards, this information can be analyzed and exploited by the own back end system. This layer implements a REST (*Representational State Transfer*) web service. This simple technology has been selected for several reasons: simplicity of implementation in both client and server side, scalability in number of clients, and reduction of interaction latency between client and server, among others.

**Figure 7:**
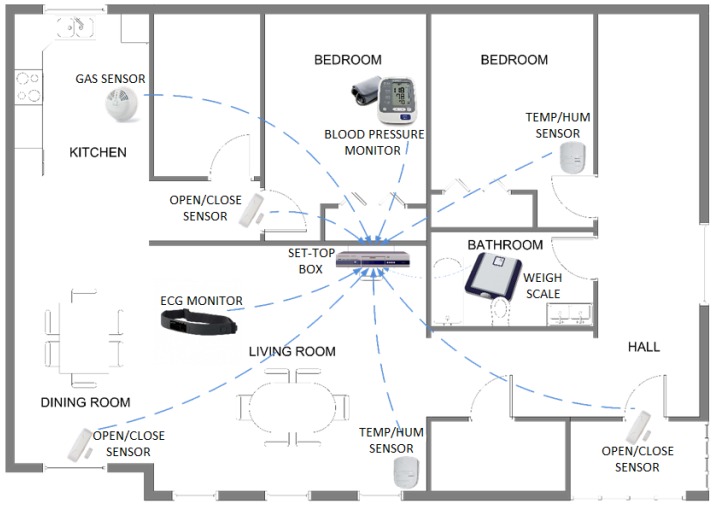
A home monitoring scenario in which a Social Sensor network is deployed.

A real monitoring scenario for evaluation purposes based on social and health sensors is depicted in Figure 7. The scenario is a home monitoring service where the user’s health and environment status is continuously acquired and received by a set-top box. The user’s information is later transmitted by this gateway device to a remote call center in order for it to be displayed, stored, or analyzed by specialist staff. The system consists of multiple acquisition sensors; some of them (portable health and social sensors) are placed at fixed locations, and others (wearable health sensors) are worn by the user. Concerning wireless communication and processing capacity features, these acquisition sensors could be implemented with no particular restriction with the evaluation module’s hardware introduced in this paper. Consequently, the real sensors should implement only a specific acquisition and conditioning block according to the type of measurement that will be taken. Some acquisition sensors that could be included in the monitoring service are described below.

- Blood pressure monitor: This health sensor is generally located in the same living space (bedroom or bathroom) and it is only used when the user must take a blood pressure measurement. This acquisition process is performed several times per day.
- Weigh scale: The performance of this sensor is similar to blood pressure monitor. It stays in the bathroom and provides weight measurements several times per day.
- Electrocardiogram monitor: This wearable sensor usually acquires the user’s electrocardiographic signal continuously, and for this reason, it is not located at a fixed position. Instead, the user wears it all the time and the acquired electrocardiographic data are transmitted to the set-top box in real-time mode.
- Temperature/Humidity sensor: This social sensor is placed in every living space for taking temperature and humidity measurements frequently (one or two measurements per minute).
- Gas sensor: This sensor is usually placed in the kitchen and takes gas measurements frequently in order to detect possible gas leaks.
- Open/close detector.

Obviously, a real scenario could include other sensors like movement detector, water consumption monitor, glucometer, pill dispenser/reminder, among others; the monitoring service will include health and social sensors according to the control requirements of the user.

In general, social sensors are characterized by enabling autonomous operation, and not requiring any specific user intervention. Sensors acquire the environment information periodically, and establish wireless communication with set-top box each time a relevant measurement is available. An important feature shown by these sensors is the possibility of configuring functional parameters like acquisition rate and transmission threshold; in this way, the sensors’ power consumption can be optimized while maintaining the transmission of outstanding environment data. The scenario includes social sensors that require different responses by the monitoring service. On the one hand, sensors like temperature and humidity monitors that do not demand any fast intervention on user’s environment if the acquired data exceed a fixed level. The system only must send the information to the call center and switch on the air conditioning unit. However, other sensors trigger a fast intervention on user’s environment when the measurement value is over the threshold because it can imply a hazardous situation to the health; gas detector is a representative example of this type of sensor.

Concerning health sensors, electrocardiogram monitor operates generally in an autonomous way. When the user switches on the sensor, it starts the acquisition and transmission of electrocardiographic signal; the sensor’s operation is completely transparent to the user. Other sensors (weigh scale, blood pressure monitor, glucose meter, *etc.*) require the user to take specific measurement steps: sensor switching on, blood pressure cuff placement, start button, wireless communication establishment, among others. In any case, all health information acquired by sensors is transmitted to the set-top box.

Independently of the sensor type, all of them must establish Bluetooth wireless communication with set-top box when there is outstanding data to transmit. Usually, the sensor will establish connection any time it must transmit information, although the sensor can also maintain an established connection permanently and transmit the information when is required. While the sensor does not have measurements to be transmitted, it can remain in low power consumption states (Park, Sniff and Hold are specific Bluetooth low power states) but the communication stays alive. Thus, set-top box receives continuously acquired data from all social and health sensors. Once the set-top box has received outstanding information, it is transmitted to the remote call center to be displayed and analyzed by specialist staff. This large-range communication can be achieved through data service of mobile phone network, although it will be carried out using Internet access with wired technologies, mainly ADSL services. This type of Internet access is widely available in society so use of these communication resources in order to send the information associated to the social-sanitary monitoring service is reasonable.

## 4. Characterization and Impact of Wireless Channel Behavior

Once the Social Sensor device has been designed and implemented and with the aim of testing system viability, wireless channel behavior has been estimated using an in-house developed 3D Ray Launching code and empirical measurements. The relevance of this test is given by the fact that wireless channels, especially in complex indoor environments lead to large signal degradation and hence, overall poor system operation. In this sense, the impact of the scenario in transceiver channel as a function of device location as well as on the obstacle density of the scenario under analysis will be tested. Estimation of propagation losses provides an estimation of received power level, which in turn can be compared with sensitivity thresholds. This way, coverage areas can be determined, which also depend on the information transmitted (*i.e.*, required bit rate and user mobility). In this way, the required node configuration can be implemented, in terms of antenna election, node placement and required number of nodes to be deployed. The simulation method has been widely tested in the literature [35,36,37,38,39] providing good results with a low computational cost.

The in-house developed 3D Ray Launching code is based on Geometrical Optics (GO) and the Uniform Theory of Diffraction (UTD). The principle method is that a bundle of rays are launched from the transmitter point with a horizontal and vertical angular resolution within a solid angle. Several transmitters can be placed within the indoor scenario. It is important to emphasize that the whole scenario is divided into a grid of cuboids, thus the parameters of the rays propagating along the space are stored in each cuboid for later computation. Parameters such as frequency of operation, radiation patterns of the antennas, number of multipath reflections and cuboid resolution are introduced. Electromagnetic phenomena such as reflection, refraction and diffraction have been also taken into account.

**Figure 8:**
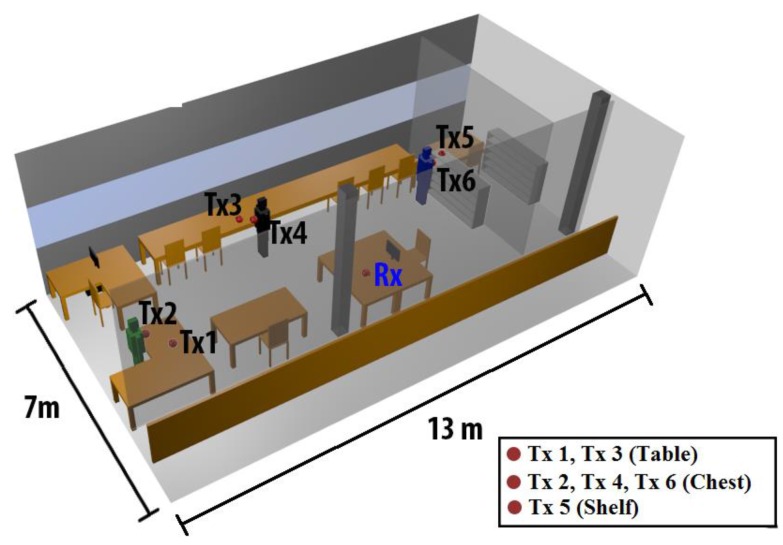
Schematic representation of the considered scenario with the three different positions of the human body and six different transmitter antenna points. The position of the receiver is also shown.

The scenario where a campaign of measurements has been deployed, depicted in Figure 8, corresponds to a typical office environment in the R&D Building of the Public University of Navarre. The scenario has been modeled three dimensionally and embedded in the simulator. All the objects and furniture within the environment have been considered, like the tables, chairs, shelves and windows, considering their material properties in terms of conductivity and dielectric constant. The size of the scenario is 13 m × 7 m × 4.2 m. In order to achieve a compromise between accuracy of the results and simulations computational time, the cuboids size and the considered number of reflections have been fixed to 0.1 m × 0.1 m × 0.1 m and 5, respectively.

In order to emulate a real situation where a wireless communication system is working with a patient in a typical indoor environment, different positions of the person and the transmitter antenna have been considered. Figure 5 shows the fixed position of the receiver, which is on the surface of a table, emulating a fixed receiver device that is in charge of recollecting the data of the patient. Three different positions of the person have been considered, as depicted in Figure 8. Besides, for each person position, two different cases for the transmitter antenna have been analyzed. The first case was with the transmitter antenna above a surface (Transmitters 1, 3 and 5) emulating a fixed transmitter nearby the person, for example, a bascule. The second case (Transmitters 2, 4 and 6) was with the transmitter devices on-body, specifically in the chest of the person, emulating an on-body device, like a pacemaker.

Real antennas have been considered for simulation, taking into account their radiation diagram pattern, polarization, transceivers gain, and transmitted power. The simulation parameters are shown in Table 2.

**Figure 9:**
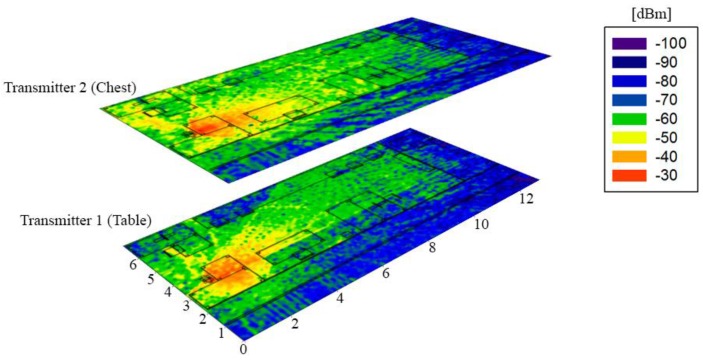
Bi-dimensional planes of Received Power (dBm) for the receiver antenna height (1 m) for two different transmitting cases, Transmitters 1 and 2.

Figure 9 shows simulation results for Transmitters 1 and 2. It shows the bi-dimensional planes of received power for the receiver antenna height (1 m). It should be pointed out that Transmitter 1 was collocated on the table and the Transmitter 2 was on-body, specifically in the chest. The radio channel complexity in both planes can be observed. On the one hand, in the case of Transmitter 1, there is a higher influence of the surface of the table in the received values, and, on the other hand, in the case of Transmitter 2, a great impact of the person is observed due to the scattering originated for the position of the antenna in the chest of the person. In addition, the influence of walls and furniture in the environment is also represented. Due to the use of transceiver elements employing Bluetooth communication, intra-system interference is not a relevant issue in the present case, due to inherent interference control in channel access of transmitters within the network. In any case, it is compulsory to meet with receiver sensitivity levels in order to guarantee adequate service as a function of transmission bit rate.

**Figure 10:**
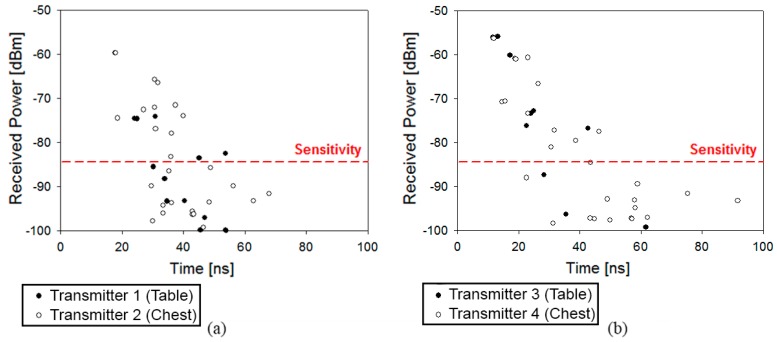
Comparison of Power Delay Profiles for both positions of the antenna (Transmitters 1 and 2): (**a**) Position 1 (green person in Figure 8); and (**b**) Position 2 (black person in Figure 8).

As can be seen in Figure 9, the impact of multipath propagation in the environment is highly important. In order to represent this effect, the Power Delay Profiles (PDPs) in the receiver point for two different positions of the person have been depicted. Figure 10 represents the PDPs for Position 1 (green person in Figure 8) and Position 2 (black person in Figure 8) for both positions of the transmitter, above the table and on-body. It can be seen that multipath behavior is absolutely vital in this type of environment. Furthermore, not only does the position of the antenna (on-body or above a surface) have a great impact in electromagnetic propagation, but also the distance between transceivers: in the case of Position 2, there are higher values of multipath received values.

**Figure 11:**
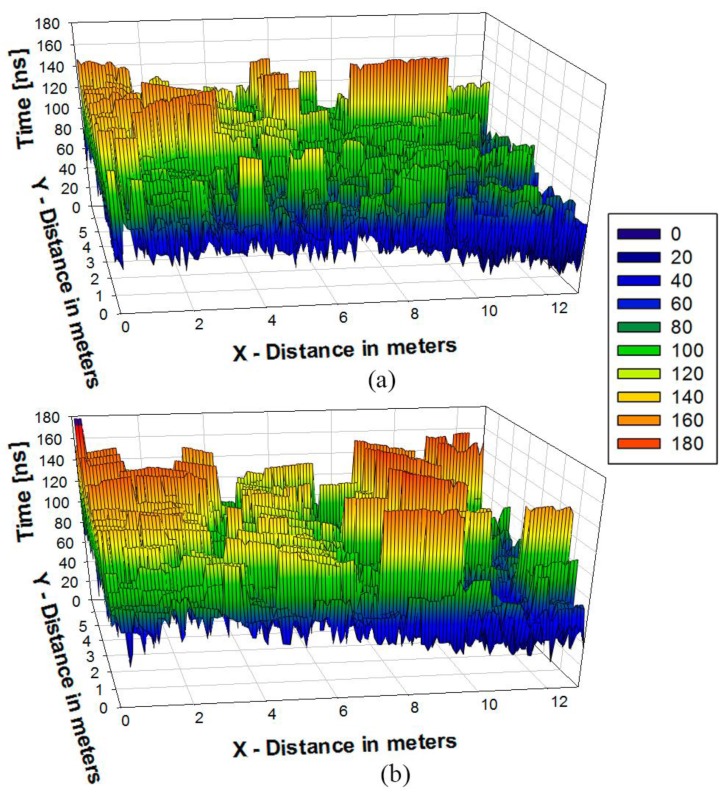
Delay Spread estimation at a bi-dimensional plane at 1 m height for Position 1 (green person in Figure 8): (**a**) Transmitter 1 (Table); and (**b**) Transmitter 2 (Chest).

The delay spread can be a good indicator of the propagation dispersion when the complexity of the scenario is elevated. In Figure 11, a Delay Spread map is depicted for the case of Transmitters 1 and 2. Higher delays are visible nearby the transmitter antenna. This is due to the fact that higher values of power are received in these points and the contribution of the reflections is higher. Nevertheless, there are some critical points such as corners or around some obstacles where the delay spread could be elevated depending on the morphology of the environment. This is the reason why the Delay Spread and Power distribution changes completely when the position of the transmitter antenna changes. The impact of delay spread variation to end-user operation is given by the fact that larger fast fading losses can decrease overall received signal levels, with values below sensitivity thresholds and hence with large error rates. Moreover, reception of multiple propagation components increases bit error rate, given mainly by Inter Symbol Interference. In the case of Bluetooth transmitters, effective transmission bit rates are low compared to operation bandwidth, implying that conventional channel equalization techniques can be employed to mitigate potential information degradation. Care, however, should be taken if transmission rate is potentially increased (*i.e.*, by using new standards) or operational frequency is decreased.

**Figure 12:**
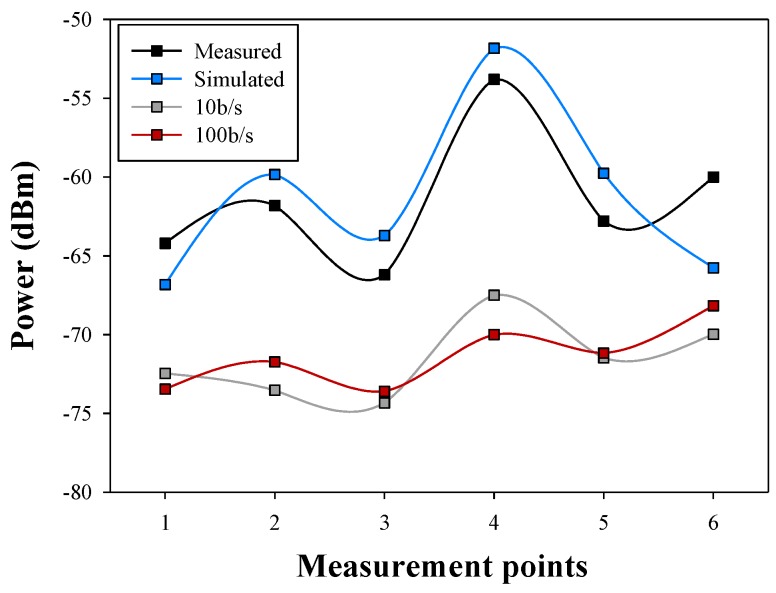
Comparison among simulated, Received Signal Strength Indication (RSSI) and measured power values.

Finally, the comparison between measurements and simulations for the aforementioned six points is depicted in Figure 12. Measurement results have been obtained with the aid of an Agilent 9912 portable spectrum analyzer, coupled to short vertical monopoles, tuned in the center of the 2.4 GHz band, consistent with Bluetooth specifications given by the employed transceivers. The spectrum analyzer data and the RSSI obtained from the device are compared and two different data rates are also considered. Good agreement between simulation and measurement data can be seen, even in the case of a complex scenario, obtaining a mean error of 0.18 dB and a standard deviation of 3.24 dB.

**Figure 13:**
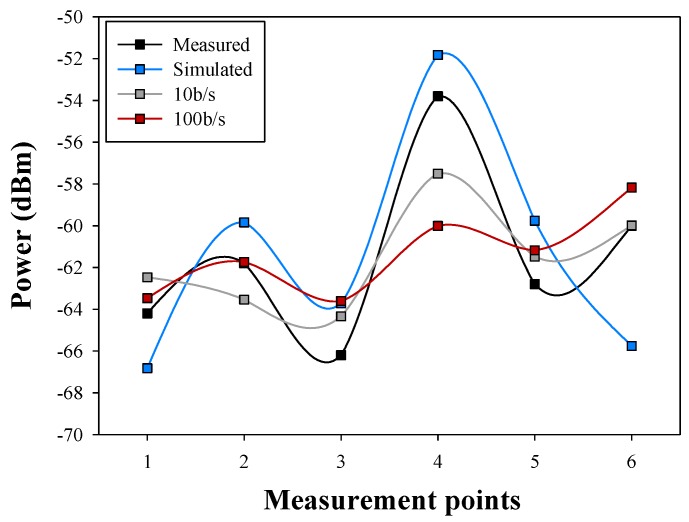
Comparison among simulated, RSSI and measured power values introducing RSSI power deviation correction.

The RSSI values usually have an error when they are compared with the spectrum analyzer caused by the kind of modulation used and therefore depending on the device used. In this case, an error of 10 dB is introduced and it is corrected in Figure 13. The existing error between corrected RSSI and the simulation is of 0.26 dB for 10 b/s and 0.07 dB for 100 b/s. The lower bit rate adjust better than the higher to the simulations power values, considering that the standard deviation in the first case is 4.1 dB and in the second case is 4.85 dB. The values that have been obtained are in all cases 15 dB above the sensitivity threshold of the employed transceivers, indicating that communication is feasible at any given location and position of the transceivers within the scenario under analysis, for the given transmitter to receiver linear radial. However, if location is changed, or if height is modified, sensitivity levels are not achieved. In the example scenario, the optimal configuration would be achieved with 3 transceivers (for the case of a relatively large room, as depicted in Figure 8), located in equidistant positions at a medium height (*i.e.*, equivalent to chest height of the user standing in the scenario).

## 5. Social Sensor System Design and Discussion

**Figure 14:**
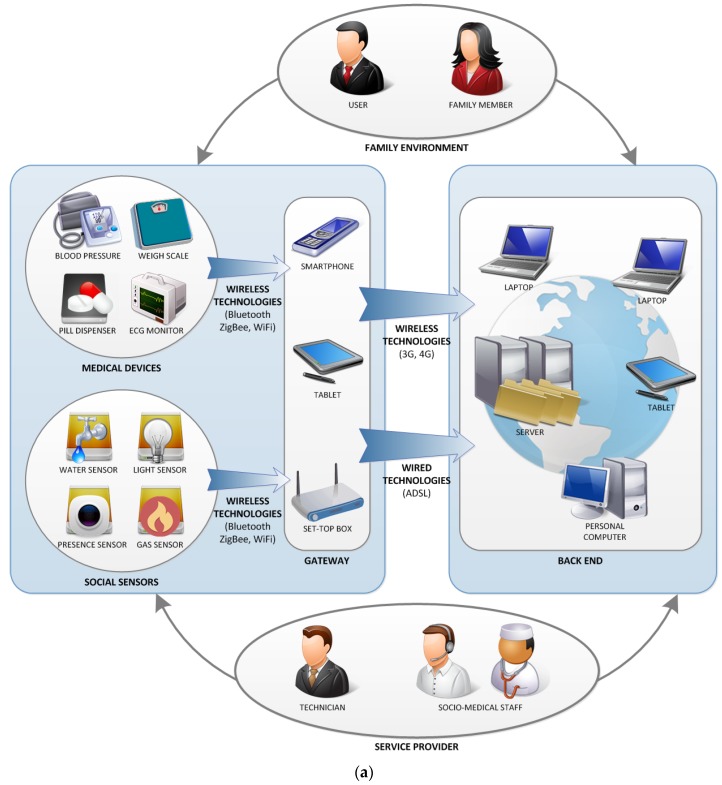

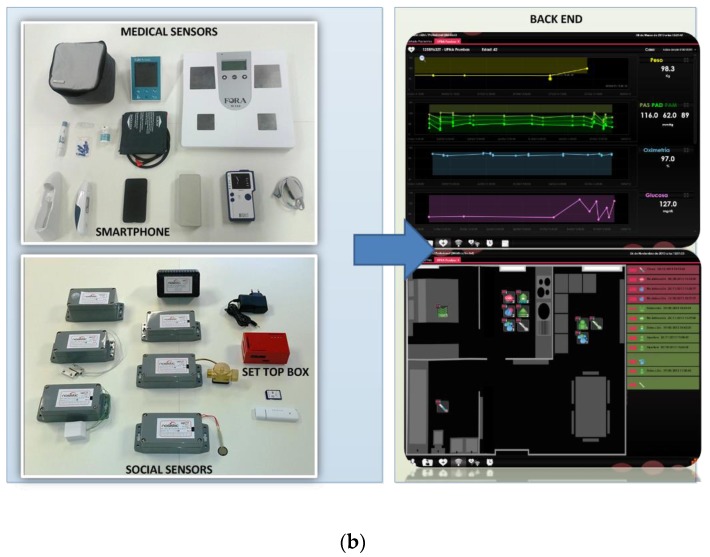
Overall view of the NASISTIC Social Sensor architecture, as well as an expanded view of the employed sensor test bed (**a**) generic architecture; (**b**) medical and social sensors together with application screenshots.

As previously stated, the solution implemented within this work has been developed under the framework of NASISTIC project, in the region of Navarra, in Spain. The NASISTIC project has been deployed in five households (dense urban area in the capital Pamplona, rural, mountain, people at risk of social exclusion and vulnerable older staff) in which medical devices such as Glucometer, Tensiometer, weigh scale, Thermometer, Control and Medication pulsioximeter as well as social sensors (humidity, temperature, presence, *etc.*), all based on the hardware-software approach described in this paper, are included. This uniformity in hardware has enabled rapid time-to-market development, reducing overall cost. A schematic description of the NASISTIC architecture, elements and back end is depicted in Figure 14.

Red Cross in Navarra has been the institution responsible for selecting the pilot users, addressing their training for the use of medical devices and taking the Call Center project. It has also been commissioned to conduct a survey of user satisfaction. In this sense, we can highlight:

- The proper functioning of the hardware proposed within the standard dimensions of a household in Spain (typically <90 m^2^ as an average value).
- The need to simplify as much as possible the use of medical devices. On the contrary, the Social Care system is almost user transparent (plug and play function). This plug and play operation for medical devices is well accepted.
- The users showed interest in participating in the pilot deployment phase. From the medical point of view, the service provides them certain levels of self-control. The users have found of particular interest knowing their blood pressure on a daily basis as a security parameter related to cardiac health. From the social point of view, the interest of users has not been as high as with medical parameters. By contrast, in this case family members (second-users) have shown interest in issues such as temperature variation in the monitored homes, flood, *etc*.
- The large amount of stored data enables the development of additional projects related to social behavioral patterns, monitoring of chronic patients with social problems such as exclusion, loneliness, energy poverty, *etc*.

The acquisition devices gather the user’s information and they can be divided into following types: medical devices and social sensors. Medical devices perform the acquisition of outstanding biomedical data in order to know the user’s health state. Some examples of medical devices are blood pressure, weigh scale, pulsioximeter, glucometer, and pill dispenser, among others. Currently, most of these devices are portable and battery powered. Due to the fact that they are used only a few times a day, these portable devices are generally placed at fixed location in the user’s residence; when a biomedical measurement is required, the user takes the medical device and uses it. However, some medical devices require continuous use in order to diagnose a disease suffered by the user. Usually, these devices are worn in the user’s body. For this reason, essential features of these medical devices are reduced size and weight, battery powered, high operation autonomy, intelligence, and wireless communication; so-called wearable devices. An example of this type of device is an electrocardiogram monitor that performs continuous acquisition and automatic detection of outstanding cardiac episodes. Social sensors gather the user’s environment information as well as data related to his/her behavior and daily activities. Some data acquired by social sensors are temperature, humidity, window and door state, gas detection, flood, and many others. The features of these sensors depend on both the acquired parameter and the location where they are placed, but in general, they are devices with very low form factor, battery powered, and high operation autonomy.

Medical devices and social sensors use short-range wireless technology in order to transmit the gathered information to a nearby Gateway system. The implemented wireless technology depends on several factors such as coverage range, data rate, power consumption, and security, among others. Currently, most systems use standard technologies such as Bluetooth, ZigBee, and WiFi, although proprietary communication technologies are also implemented in some cases for getting high levels of optimization, data rate and/or power consumption.

Gateway device performs bridge functions between acquisition devices and back-end system. Mainly, this device receives the acquired information and transmits it to the back-end. The Gateway device can also perform additional tasks: data pre-processing and temporary storage. The first one makes it possible to obtain outstanding information about user’s state and system operation prior to the back-end system processing; it also allows the optimization of wirelessly transmitted data in order to reduce power consumption and cost. The second one prevents the system from losing data in case of communication or coverage failures.

The gateway device can be implemented in different platforms such as smartphone, tablet, or set-top box. All these platforms use short-range wireless technologies to communicate with acquisition devices. However, the data exchange between Gateway and back-end system can be performed with different technologies according to platform and availability of Internet access at user’s household. Thus, mobile platforms (smartphones and tablets) will use 3G/4G technology provided by the mobile phone network; this guarantees a total coverage both inside and outside user’s household during the monitoring process. In the case of set-top box platform, the technology depends on whether the user has ADSL or cable Internet service. If Internet service is provided, set-top box can use it directly for data transmission to the back-end system. Otherwise, the set-top box must incorporate its own 3G/4G technology by means of wireless dongle or similar device.

Finally, the back-end system achieves the user’s data reception. It is made up of some servers and a database management system in order to store all the system’s information: gathered data, agents, access profiles, authentication and credentials, *etc*. The back-end system provides a web service that allows the agents to access it with their personal devices.

Several agents can be involved in a social sensor system. On the one hand, user and family members can interact with acquisition sensors and Gateway device in order to start or modify system operation; the level of this interaction depends mainly on user/family member technical knowledge. The back-end system can also be accessed remotely by the user and family members to perform several tasks: management and visualization of gathered data, reception of medical/social staff notifications, question suggestions, among others. On the other hand, medical/social staff can also access the back-end system in order to analyze and visualize the user’s information. Depending on this information and automatic alerts generated by the own system, the agent can know the user’ state and even determine behavioral patterns that must be corrected. In these cases, the medical/social staff will make contact with the user or family member in order to notify behavioral advices, medication prescription, *etc*. Finally, technical workers are in charge of installing and configuring the system operation. These agents also have maintenance functions when some system malfunction has occurred. There will be problems whose solution will be performed remotely from the service provider, but other ones will require the movement of technical worker to the user’s household; device malfunction, system configuration failure, and wrong device communication are common problems that cannot be solved by users or family members, so a technician must do it in person.

Centralized and secure management in the deployment of Socio-Sanitary services such as NASISTIC is a mandatory requirement. These back-end tools are designed for managing users with different roles (end-users, family users or secondary users, stakeholder users, *etc*.) as well as the management of information received from all monitored houses. Obviously, the end-users and their family users can access their own information. In the case of stakeholder users, access will be for all homes or end-users of their institution. In any case, these tools provide a complete configurability of roles.

Furthermore, in order to ease as far as possible the service configuration, tools like NASISTIC provide graphical aids such as either 2D or 3D drawings of housing for sensor placement, whether they are social sensors or health sensors, and tools for viewing the received data (time graphs, *etc.*). Moreover, besides a user-friendly configuration wizard, tools for configuring alerts from the received measurements are also implemented. For example, for a user, maximum and minimum blood pressure levels can be set (relative or absolute in %) or any other health sensor and alerts on social sensors like temperature (energy poverty, *etc.*) or moisture. These alerts generate messages (SMS, emails, WhatsApp messages, *etc.*) or phone calls to those in charge of the user who previously have been configured (family-users, physicians, stakeholder users or even end users).

In order to provide insight into the detailed operation of the system and to effectively test its suitability as an AAL system [40], examples of multiple signals available have been tested in a Living Lab environment, within the Public University of Navarra. As was previously mentioned, the evaluation module could emulate the functional operation of any monitoring sensor, being only required the implementation of analogue acquisition block. In this way, it is possible to emulate the home monitoring service’s operation as the system was implemented in a real scenario. For this emulation, we consider four sensors: temperature/humidity sensor, electrocardiogram monitor, weigh scale, and open/close detector. Table 3 shows the main emulated features of sensors. The gateway device is also emulated with an evaluation module that gathers the acquired information and transmits it to Laptop via RS-232 serial communication. Laptop does not implement a display software application, so the raw data are stored in a stream file for later analyze and graphic representation.

**Figure 15:**
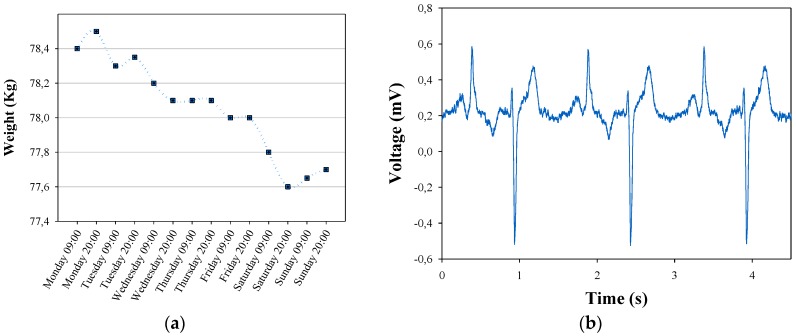

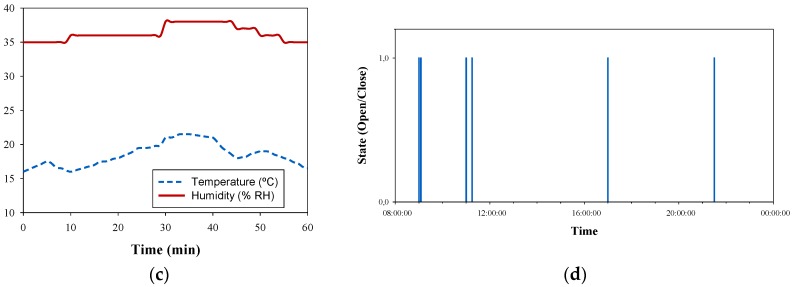
(**a**) Weight measurements obtained from the Social Sensor network devices. (**b**) Electrocardiographic signal obtained from the Social Sensor network devices. (**c**) Temperature and relative humidity measurements. (**d**) Hall door opening and closing events.

As an example, Figure 15a,b shows measurements related to weigh scale and electrocardiogram monitor devices, respectively. The first one shows the user’s weigh scale evolution throughout a week. This evolution, if it were to continue for too long, could be representative of a poor diet in ageing people or even suffering a neurodegenerative disease like Alzheimer’s disease. The second figure shows five seconds of user’s electrocardiographic signal. This health information is not usually analyzed by the user but instead a cardiologist in order to verify the heart activity and diagnose possible cardiac diseases, such as ventricular arrhythmias, atrial fibrillation, *etc.*

With regards to social sensors, temperature and relative humidity measurements are depicted in Figure 15c. As can be seen, temperature values range from 16 °C to 19 °C, and relative humidity from 35% to 38%, which could be usual in a home monitoring system. These measurements give the user and service provider relevant information about user’s comfort and environment state during daily life conditions. Once again, ambient temperature and humidity values out of suitable comfort ranges could be representative of *Heating-Ventilation-Air-Conditioning* (HAVC) system malfunction and/or its inappropriate control by the user. Finally, Figure 15d shows the number of times the hall door is opened/closed and how many time it has remained in each state. Although it does not provide specific user’s information, its analysis could be useful for inferring behavior aspects and daily activities. In this case, it is possible to know the time when the user has left from/arrived to home, or some people have visited the user; the information even allows knowing the time period the hall door has stayed opened.

**Figure 16:**
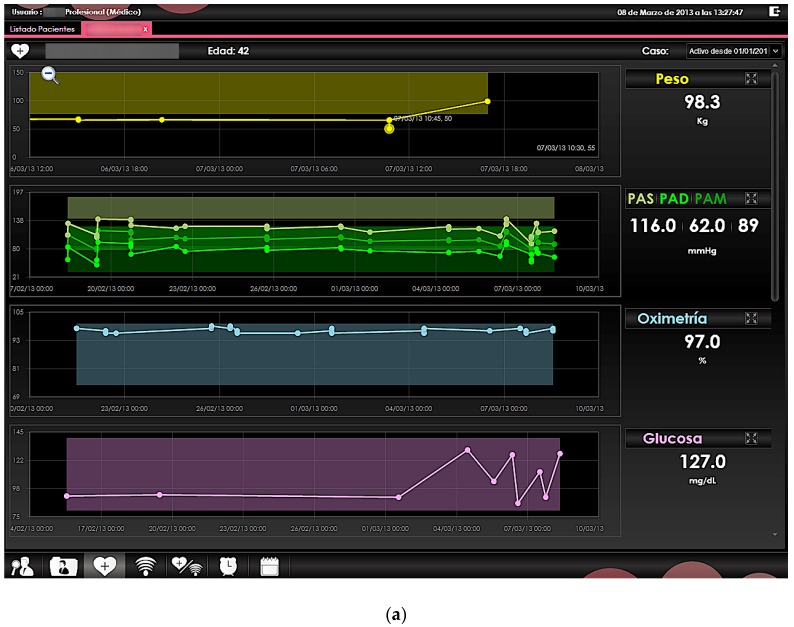

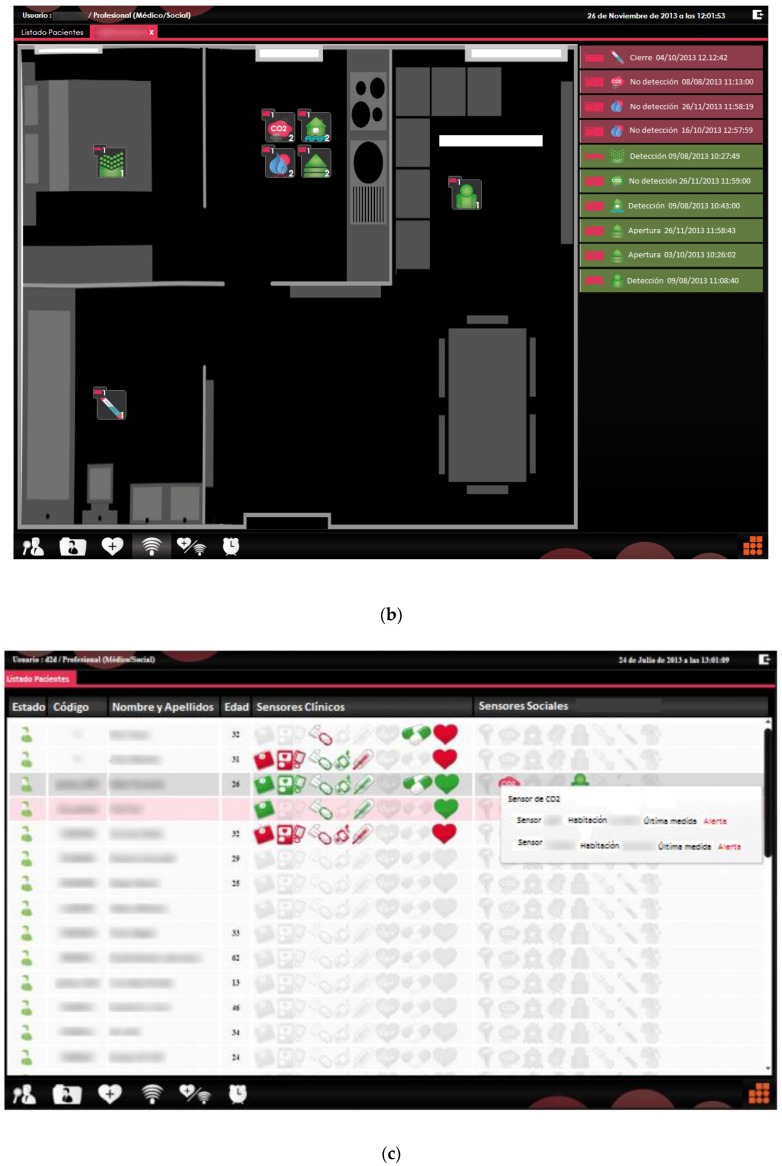
View of the application layer implemented in the back-end of the NASISTIC system. A view of different sensor signals, location map and message alerts are depicted (**a**) Sensor signals. (**b**) Location map of sensors. (**c**) Sensors and alerts associated to users.

As previously stated, the information gathered by the social sensors and the health sensors are processed by the back-end servers, where database processing as well user interfaces are implemented and managed. Figure 16 shows the implemented application layer, in which information such as monitoring of social sensors and health sensors, location of sensors within the household or detailed view of existing alarms can be obtained. The system is designed in order to provide a modular and easily accessible screen configuration to access relevant information, such as bio medical signal status and alarm monitors.

The system has been developed in order to minimize the operation problems. In any case, the following are the main reasons for malfunction and detection mechanisms provided by the system to correct them.

- Device malfunction or misuse: At any time, a medical device or social sensor can show a wrong operation; even when that device has a proper operation, it can be misused by the user or caregiver. The service provider staff must detect these situations in order to solve the problem, by either replacing the broken device or retraining the user/caregiver on the correct use. Thus, the NASISTIC system includes a log message module that registers the main activity performed by devices. These messages are transmitted to the back-end so they can be viewed and analyzed by technical worker.
- False notifications: The system allows the configuration of alerts based on the received measurements in order to generate messages/notifications to those in charge of the user. During system operation, some occasional wrong measurement can be taken due to device malfunction/misuse or unusual environment conditions, which will generate false alerts. Obviously, there are acquisition parameters (e.g., temperature and humidity) that allow analyzing measurement trends in order to detect occasional out of range data. In this way, the system rejects the data and does not generate a false alert. Other acquisition parameters (e.g., gas, open-close, flood) require generating an alert in any case the measurement is out of range because it implies a hazardous situation. Currently, the NASISTIC system does not implement any strategy for distinguishing occasional out of range data with no risk for the user; all alerts lead to warning notifications.

Some problems were registered during the deployment of NASISTIC system. On the one hand, operation failures because of wrong system configuration. Devices configured with erroneous network address, sensors set with inappropriate operation features as device type, measurement identification, or date/time reference, and back end’ server address not configured in the gateway device were the most usual mistakes. Problems related to erroneous configuration of sensor’s features were detected by means of log messages, while those ones related to wrong setting of communication parameters were detected simply with no reception of measurements after a time the system was operating. In any case, the technician inspected the system in the user’s residence and reconfigured it in order to guarantee the correct operation.

On the other hand, limited number of false alerts originated in medical device misuse; body temperature not registered correctly due to inadequate contact between skin and thermometer was the most relevant problem. These false alerts caused the corresponding calls to the user/caregiver in order to notify him or her about the event and remind him or her how to correctly use the medical device.

In general, the system has been developed with the aim of reaching most social monitoring situations. Certainly, the correct use of the system depends on several relevant factors. On the one hand, the user or caregiver/family member must have a basic knowledge of medical devices use. In those social situations where this requirement is not met, the monitoring system cannot be used; the number of these situations is limited but not zero. On the other hand, the user’s household must be located in a region where Internet access and/or 3G wireless coverage are not limited. If this requirement is not fulfilled, the communication between Gateway device and back-end system is impossible and thus the user cannot receive follow up care. These situations are also limited, although users living in isolated regions or mountain villages can suffer from this problem.

In order to provide a holistic view of the implemented solution, an initial assessment from end users as well as from medical/social professionals involved in initial NASISTIC trials reveals the following statements:

- The proper operation of the proposed hardware is possible within the standard dimensions of a household in Spain (typically <90 m^2^ as an average value). Although there are many factors involved in the wireless communication between devices, the use of Bluetooth Class 2 technology guarantees a suitable coverage range of approximately 10 m. This communication range, together with the set-top box usually being placed in the house’s central room, makes it possible to use the proposed system in most households.
- The need to simplify as much as possible the use of sensors and devices. In the case of social system, the sensor’s operation is almost user transparent because they only require being one time configured and switched on; this plug and play operation is well accepted. In the same way, the use of medical devices must be as simple as possible in order to guarantee the user/caregiver approval, and reduce possible measurement errors. It must be taken in account that end user likely show low technology knowledge, and any additional required intervention in device operation can make the user reject the system; even in the case of a medical device being used by the caregiver, a complex device operation can lead to wrong measurement acquisition, incorrect function selection, or data transmission fail. For this reason, and because most of the medical devices require some user intervention (press button, cuff placing, *etc.*), it is necessary to minimize the number of these actions. All these considerations have been taken into account in NASISTIC project development.
- The users and social/medical staff have shown significant interest in participating in the NASISTIC pilot deployment phase. With regards to users, personal interviews between them and Red Cross assistants have derived some first conclusions. On the one hand, they accept the use of proposed system, which is perceived as an additional element within the household. On the other hand, they have found of particular interest knowing some biomedical parameters (blood pressure mainly) on a daily basis as security data related to health state. From the social point of view, the interest of users has not been as high as with medical parameters; in contrast, in this case, family members (second users) have shown interest in issues such as temperature variation in the monitored homes, flood, *etc*. With regards to social/medical staff, the main results obtained are the following: response times are reduced due to real time interaction capabilities offered by the social sensors, and the general service provides them certain levels of self-control. Although specific survey forms have not been implemented, the daily personal contact between users and social/medical staff during the project deployment has made obtaining these first satisfaction data possible.
- The large amount of stored data enables the development of additional projects related to social behavioral patterns. Meanwhile, biomedical data allow the knowledge of user’s health state and mood, while other activity data, such as watching TV, use of HVAC system, water consumption, or length of phone calls, can provide information about user’s behavior and social relationships. All this information can be combined with user activity on the Internet and specific web social networks like Facebook, Twitter, *etc.* This makes broader monitoring applications focused on following up with users with chronic social problems such as exclusion, loneliness, energy poverty, *etc.* possible.
- Technical workers, engineers and architects involved in the deployment indicate that the proposed system is simple to integrate and maintain, reducing overall installation and operational costs. Thus, start-up system requires minimum intervention by technical worker because elements are pre-configured from factory or service provider; the worker only has to verify the correctness of system operation the first installation time. In addition, the system maintenance neither demands a great number of home interventions by engineers or technical workers unless some element must be repaired or replaced.

## 6. Conclusions

In this work, the implementation of Context Aware in order to aid in the assistance of end users within the framework of e-Health and m-Health scenarios has been described. A prototype of Social Sensor device has been designed and implemented in order to provide multiple-signal processing capabilities, while exhibiting flexible, low complexity and moderate cost features. The Social Sensor device is based on a Bluetooth transceiver, in which a dedicated microcontroller provides access to several signals, which can provide medical, environmental or behavioral information. In order to analyze the robustness of the system, detailed wireless channel analysis has been performed, providing insight on the behavior of the devices in real complex indoor scenarios, in which large signal variability can severely degrade overall performance. Estimations of received power levels have been compared with experimental results, indicating that received power levels comply with receiver sensitivity levels in the scenario for any potential transceiver location, implying adequate overall system service levels. Initial tests under Living Lab conditions, given by NASISTIC operational requirements have also been performed, showing the feasibility to successfully interchange multiple information within real time regime. Trial runs have shown the feasibility in data acquisition of multiple bio-physical parameters as well as user information, leading to a practical AAL platform. The proposed solution is currently being prepared for large-scale deployment because of the ease in installation and maintenance, positive adoption by end users and adequate results in terms of response time reduction and preventive actions that can be adopted.

The NASISTIC system is a remote health and behavior monitoring system, providing means to health professionals as well as social services to provide increased quality of living to vulnerable population segments, such as elderly people, and those suffering from some form of disability or mental illness. The difference between NASISTIC and other proposed systems is the integral approach in capturing biomedical signals as well as other signals that are related with user habits, such as usage of lights, heating, electrical appliances, water consumption or open/close indication of doors. In this way, combined action of health specialists and social services can be provided in order to enable higher degrees of autonomy to the users as well as increasing quality of life levels.

As future steps, complete system validation, integration of additional sensor elements, and the implementation of a distributed system architecture based on cloud integration will be performed. The current implementation of NASISTIC system uses a back-end server for information storage. Although this first version is based on a centralized solution, the system is not limited to it. Suitable modifications on back-end element can lead to distributed implementations based on cloud solutions.

Additionally, complete user experience assessment will be obtained in order to identify usability, leading to further modifications and enhancements.

## Figures and Tables

**Table 1:** Health monitoring system comparison.

System	Transmission System	Architecture	Application Scenario	Detected Variables	Reference
LiveNET	PDA Connectivity	Centralized	Detection of Epilepsy Seizure, Parkinson symptom detection, Soldier Health Monitoring	3D Accelerometer, ECG, EMG, galvanic skin conductance	[25]
AMON	Wrist bracelet, GSM	Operator Based	Estimation of Patient Health Conditions	Blood Pressure, Blood Saturation, Skin Temperature, ECG	[26]
LifeGuard	Bluetooth to a base station	Centralized	Multiparameter Wearable Monitoring System	ECG, respiration rate, heart rate, oxygen saturation, body temperature, blood pressure, body movement.	[28]
Real Time Wireless Physiological Monitoring System	Low Power Cordless Phone To Base Station	Centralized	System Aid in Nursing Centers and Hospitals	Blood Pressure, Heart Rate and Temperature	[27]
Brain Injury Monitoring System	Bluetooth to Home PC	Centralized	Monitoring of Brain Injured Infants	Blood Saturation, Heart Rate, Respiration, Body Movement.	[29]
MyHeart	Communication to Data Logger	Off-line	Wearable System for Heart Disease Monitoring. Sensors knitted or embedded in garment.	ECG, Activity Sensor.	[30]
Wearable Health Care System (WEALTHY)	Bluetooth/GPRS	Centralized or Datalogger	Application to clinical patients during rehabilitation, elderly people, patients with chronic diseases	ECG, EMG, thoracic and abdominal respiration rate, body position, movement	[31]
MagIC	Bluetooth	Centralized	Woven textile sensors in a washable vest	ECG, respiration rate, motion level.	[32]
Medical Remote Monitoring of Clothes (MERMOTH)	RF Link to PDA	Data Logger	Wearable and Stretchable Sensing Garment	ECG, respiratory inductance plethysmography, skin temperature, activity.	[33]
NASISTIC	Bluetooth/2G-4G Connection	Locally Distributed/Centralized	Social Sensor Node Deployments	Combination of Biophysical signals (ECG) with user habits.	

**Table 2:** Simulation transmitter antenna characteristics.

Parameters in the Ray Launching Simulation	
Frequency	2.43 GHz
Transmitter power	0 dBm
Antenna gain	−1 dBi
Horizontal plane angle resolution (∆Φ)	1°
Vertical plane angle resolution (∆θ)	1°
Reflections	5

**Table 3:** Features of emulated health and social sensors.

Sensor	Location	Accuracy	Transmission Rate	Range	Acquisition Rate
Temperature/Humidity	Living room	±0.5 °C ±1% RH	1 value/min	0 °C–65 °C	2 samples/s
Weigh scale	Bathroom	±100 gr.	2 value/day	0 gr–150 gr	2 sample/d
Electrocardiogram monitor	User’s body	±45 µV/LSB	Real-time	−1V–2V	500 samples/s
Open/close	Hall door	-	-	0V–1V	-
